# Abscisic acid signaling regulates primary plasmodesmata density for plant cell-to-cell communication

**DOI:** 10.1126/sciadv.adr8298

**Published:** 2025-05-07

**Authors:** Chiyo Jinno, Ken Fujisaki, Izumi Yotsui, Motoki Ouchi, Prerna Singh, Satoshi Naramoto, Daisuke Takezawa, Yoichi Sakata, Tomomichi Fujita

**Affiliations:** ^1^ Faculty of Science, Hokkaido University, Sapporo, Hokkaido 060-0810, Japan.; ^2^ Department of Bioscience, Tokyo University of Agriculture, Tokyo 156-8502, Japan.; ^3^ Graduate School of Science and Engineering, Saitama University, Saitama 338-8570, Japan.; ^4^ Graduate School of Life Science, Hokkaido University, Sapporo, Hokkaido 060-0810, Japan.; ^5^ JST-PRESTO, 4-1-8, Honcho, Kawaguchi, Saitama 332-0012, Japan.

## Abstract

Cell-to-cell communication is essential for multicellular organisms. Plasmodesmata (PD) are plant-specific nanopore structures pivotal for cell-to-cell communication and plant survival. However, how PD form and their structure, regulation, and evolution remain largely unknown. Here, we demonstrate that the exogenous supply of abscisic acid (ABA), a well-conserved phytohormone in land plants, reduces primary PD density in the moss *Physcomitrium patens*. This regulation requires all core components of the ABA signaling pathway. Furthermore, we reveal that ABA-INSENSITIVE 5, a well-conserved transcription factor in the ABA signaling pathway of land plants, plays a pivotal role in PD density regulation, whereas ABA-INSENSITIVE 3 does not. Our findings show that the ABA-induced reduction in primary PD density is mediated by these ABA-responsive factors in *P. patens*. Considering previous reports on ABA-dependent PD regulation in both moss and angiosperms, we propose that the ABA-mediated control of PD biogenesis and permeability represents a conserved mechanism in land plants, with critical implications for cell-to-cell communication and stress adaptation.

## Teaser

Abscisic acid reduces primary plasmodesmata density in moss through conserved ABA signaling factors of land plants.

## INTRODUCTION

Regulation of cell-to-cell communication is essential for the growth and environmental responses of multicellular organisms. In animals, tetraspan integral membrane proteins termed connexins play a pivotal role in this process by forming and regulating direct intercellular channels known as gap junctions, which bridge plasma membranes between adjacent cells, affecting cell proliferation, migration, and tumor growth (*1*–*3*). In contrast, plants (land plants or embryophytes), another major group of multicellular organisms, have developed distinctive symplastic channels called plasmodesmata (PD) (*4*–*7*). PD have been observed in the Charophyte alga orders Charales and Coleochaetales and are considered to have originated from the common ancestor of current Phragmoplastophyta species; meanwhile, the complete loss of PD and partial loss of the phragmoplast in Zygnematales, as well as the structural differences in PD among different lineages, have led to an alternative hypothesis that PD evolved independently multiple times within Phragmoplastophyta species (*8*–*11*). The most common hypothesis for PD formation was first described on the basis of electron microscopy observation: The endoplasmic reticulum (ER) meshwork is trapped in the newly forming cell plate during cell division, and plasma membrane–lined holes are maintained in that area, which become primary PD (*12*–*14*). For more than half of a century since then, the molecular mechanisms underlying primary PD formation and the regulation of PD number or density during cytokinesis have largely remained a mystery, in contrast to some aspects of secondary PD biogenesis, where secondary PD are formed in existing cell walls between cells (cross walls) (*15*–*17*). In *Arabidopsis thaliana*, genetic studies have uncovered several proteins affecting secondary PD formation; however, the impact of these factors on secondary PD formation is thought to be indirect because none of them localize to PD (*18*–*21*). While several phytohormones have been reported to regulate PD (*22*–*24*), recent studies have identified abscisic acid (ABA) as one of the key regulators (*25*–*28*). The physiological functions of ABA are well established; it is essential for various abiotic stress responses, including drought, high salinity, and low temperatures. ABA plays a pivotal role in enabling sessile land plants to survive water-limited stress conditions by regulating the expression of stress response genes (*29*, *30*).

## RESULTS

### Long-term ABA treatment affects PD density in the newly formed cell walls of brood cells

Studies over the past few decades have identified the core components of ABA signaling pathway, consisting of an ABA receptor family PYR/PYL/RCAR (PYRABACTIN RESISTANCE/PYRABACTIN RESISTANCE–LIKE/REGULATORY COMPONENT of ABA RECEPTOR), SUCROSE NONFERMENTING 1–RELATED PROTEIN KINASE 2 (SnRK2), and the type 2C protein phosphatase (PP2C), ABA-INSENSITIVE 1 (ABI1), which are evolutionarily conserved among land plants (*29*, *31*, *32*). Recently, we found that PD permeability decreases rapidly in the filamentous protonemata tissue of the moss *Physcomitrium patens* (formerly called *Physcomitrella patens*) without changes in PD density within 1 hour after ABA treatment (*25*, *26*, *33*). In some mosses, prolonged ABA treatment or environmental stress (for more than a few days) causes normal protonemal cells to become brood cells or brachycytes, a special group of stress-tolerant stem cells characterized by a round cell shape with loss of cell polarity and cell adhesion, and thickened cell walls (*34*–*36*). We observed that cell-to-cell communication was markedly suppressed among brood cells in *P. patens* through an unknown mechanism (*33*). We therefore reasoned that PD structure or density might be altered in brood cells.

**Fig. 1.**
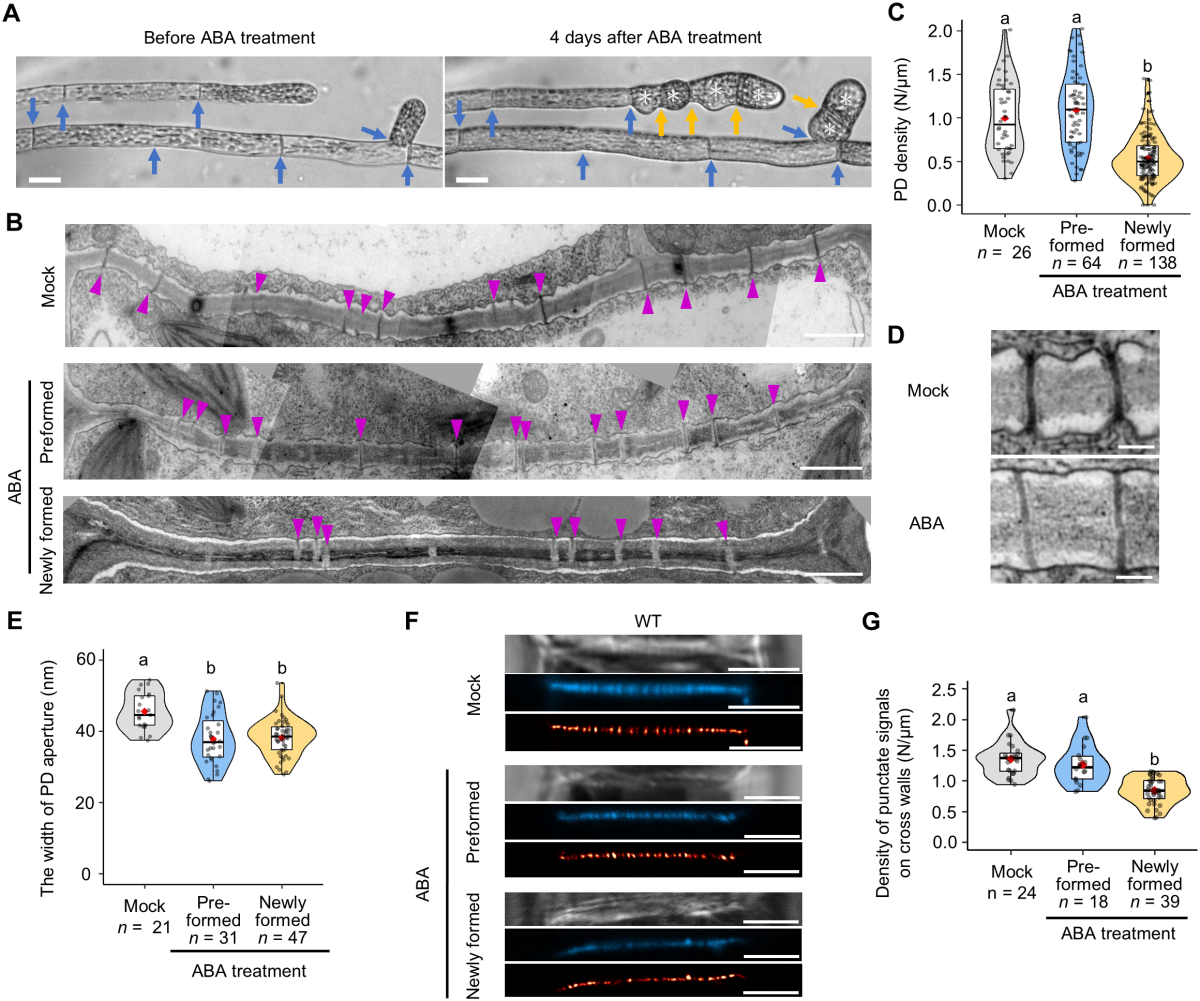
ABA reduces PD density in the moss *P. patens.* (**A**) Brood cell differentiation upon ABA treatment. WT protonemal cells before (left) and 4 days after ABA application (right). White asterisks indicate brood cells. Blue and yellow arrows indicate cross walls formed before and after ABA application, respectively. Scale bars, 15 μm. (**B**) TEM images of cross walls in mock-treated WT protonema and cross walls preformed before and newly formed after ABA application. Magenta arrowheads indicate PD on cross walls. Scale bars, 1 μm. Individual images have been manually stitched together to show the entire sample. (**C**) Quantification of PD density in WT cross walls following mock treatment and cross walls preformed before and newly formed after ABA application. (**D**) PD structure in cross walls following mock or ABA treatment. Scale bars, 200 nm. (**E**) Quantification of the width of PD apertures in cross walls under mock treatment and in cross walls preformed before and newly formed after ABA application. (**F**) Bright-field (top), aniline blue fluorochrome (middle), and super-resolution radial fluctuation (SRRF) image (bottom) of cross walls in WT under mock treatment and cross walls preformed before and newly formed after 50 μM ABA application. Scale bars, 5 μm. (**G**) Quantification of the density of aniline blue fluorescent spots in cross walls formed under mock treatment and in cross walls preformed before and newly formed after ABA application. (C, E, and G) The value of *n* indicates the number of different cross walls. One-way analysis of variance (ANOVA) followed by Tukey’s post hoc test, *P* < 0.01. Violin plots show numerical data. Box boundaries indicate the upper (75th percentile) and lower (25th percentile) quartiles, and the whiskers show 75th percentile + 1.5 interquartile range (IQR) and 25th percentile – 1.5 IQR. Median and mean are indicated by a bold line and a red diamond, respectively.

We first examined PD by transmission electron microscopy (TEM) of cross walls of the wild-type (WT) protonema treated with ABA for 4 days, where brood cells were induced (Fig. 1A, white asterisks). The PD density of these protonemata was decreased to an unexpected degree, to half that of mock-treated controls (fig. S1A). It was unclear whether this difference resulted from the degradation of preexisting PD in cross walls formed before ABA application or a reduction in de novo PD formation in cross walls formed after ABA application. Therefore, we next examined PD density in each type of cross walls—those formed before ABA application and those newly formed during 4 days of ABA treatment—by tracking the same individual protonemal filament (Fig. 1A, blue and yellow arrows; cross walls formed before ABA application and newly formed cross walls after ABA application, respectively). The shape of the protonemal cells and the position of the cross walls were recorded using bright-field microscopy. This positional information was accurately retained during the preparation of sections, allowing us to distinguish cross walls after the fixation and embedding processes were completed. The PD density in preformed cross walls that existed before treatment was similar following ABA application or mock treatment (Fig. 1, B and C; mean PD density “Mock” versus “ABA preformed” = 0.992/μm versus 1.087/μm, *P* = 0.2635). In contrast, we observed lower PD density in cross walls that were newly formed after ABA application than in preformed cross walls after ABA application (Fig. 1C; mean PD density “Preformed” versus “Newly formed” = 1.087/μm versus 0.539/μm, *P* = 1.798 × 10^–14^). These results suggest that prolonged ABA treatment diminishes PD formation in nascent cell walls.

Investigation of PD structure following prolonged ABA treatment revealed no complex or branched PD structures; all PD exhibited a simple structure (Fig. 1D). Because brief ABA treatment reduces PD permeability by narrowing the PD aperture (*25*), we compared the width of PD apertures in preformed and newly formed cross walls following extended ABA treatment. Consistent with our previous results, we observed narrower PD apertures in both types of cross walls under ABA treatment than in cross walls under mock treatment (Fig. 1E; mean PD width “Mock” versus “ABA preformed” = 45.5 nm versus 37.8 nm, *P* = 4.236 × 10^–5^; “Mock” versus “ABA newly formed” = 45.5 nm versus 38.2 nm, *P* = 5.181 × 10^–6^), indicating that long-term ABA treatment suppresses cell-to-cell communication by narrowing PD apertures in addition to reducing PD density. Thus, in brood cells, which are stress-resistant cells, a stronger inhibition of cell-to-cell communication through reduced PD density, coupled with the down-regulation of PD permeability, may facilitate their separation from one another, thereby enabling them to function as diaspores (*34*).

To examine PD density changes in response to ABA concentrations from 20 to 100 μM, we next performed aniline blue staining of PD-associated callose accumulation (fig. S1B) (*26*, *37*). We observed lower PD-associated callose density in cross walls newly formed after application of 50 μM ABA than in preformed cross walls after ABA treatment or those under mock treatment (Fig. 1, F and G; mean punctate signal density “Mock” versus “ABA newly formed” = 1.352/μm versus 0.838/μm, *P* = 2.226 × 10^–9^), which is consistent with the reduction in PD density observed by TEM (Fig. 1, C and G). The density of punctate signals observed with aniline blue staining is relatively higher than that observed via TEM (e.g., Fig. 1, C and G). This is likely due to the greater optical section thickness in the former compared to the ultrathin sections used in TEM (see the “Aniline blue staining” section in Materials and Methods). This methodological difference does not affect the comparison of PD density when the same method is applied. We further investigated PD density under different concentrations of ABA using aniline blue staining (fig. S1C), and it revealed a decreasing trend in the punctate signal density with increasing ABA, which reached a plateau beyond 50 μM (fig. S1D). Thus, these results suggest that the regulation of PD density by ABA is dose dependent.

**Fig. 2.**
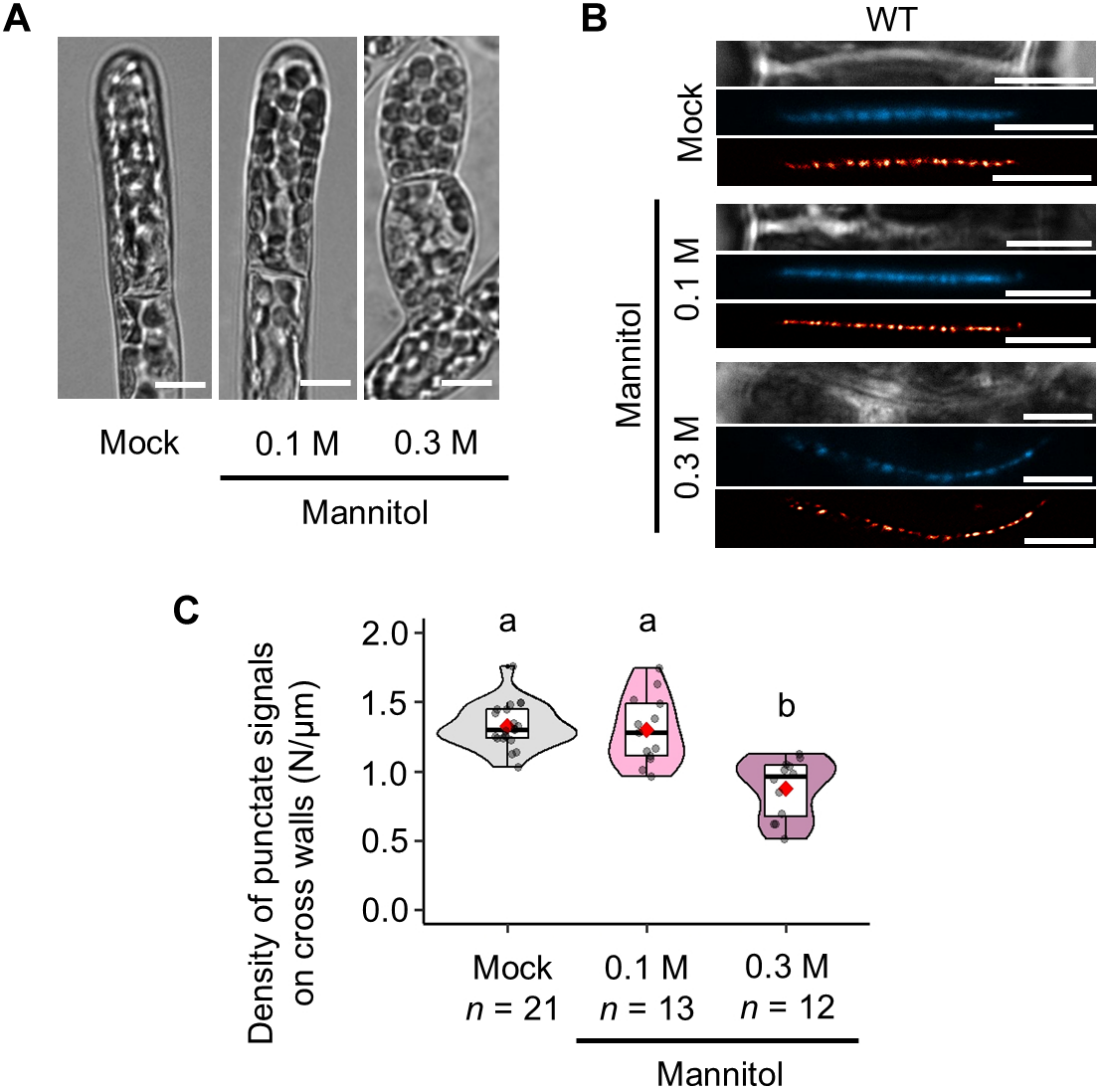
Osmotic stress reduces PD density in the moss *P. patens.* (**A**) Protonemal cells of the WT after 7 days of treatment with 0.1% dimethyl sulfoxide (DMSO) (mock), 0.1 M mannitol, or 0.3 M mannitol. Scale bars, 15 μm. (**B**) Bright-field (top), aniline blue fluorochrome (middle), and SRRF image (bottom) of cross walls in the WT after 7 days of treatment with 0.1% DMSO (mock), 0.1 M mannitol, or 0.3 M mannitol. Scale bars, 5 μm. (**C**) Quantification of the density of aniline blue fluorescent spots in cross walls of the WT following 7 days of mock, 0.1 M mannitol, or 0.3 M mannitol treatment. The value of *n* indicates the number of different cross walls. One-way ANOVA followed by Tukey’s post hoc test, *P* < 0.01. Violin plots show numerical data. Box boundaries indicate the upper (75th percentile) and lower (25th percentile) quartiles, and the whiskers show 75th percentile + 1.5 IQR and 25th percentile – 1.5 IQR. Median and mean are indicated by a bold line and a red diamond, respectively.

ABA is a crucial phytohormone in the abiotic stress response. Osmotic stress increases endogenous ABA levels and triggers a stress response mediated by SnRK2 in *P. patens* (*38*). We therefore observed the punctate signal density of aniline blue in the WT following treatment with mannitol; 0.3 M mannitol, but not 0.1 M mannitol, induced the formation of brood cell–like cells with a round cell shape (Fig. 2A) and a punctate signal density lower than that in mock-treated cells (Fig. 2, B and C; mean punctate signal density in WT “Mock” versus “0.3 M mannitol” = 1.327/μm versus 0.878/μm, *P* = 5.354 × 10^–6^). Thus, the data suggest that PD density decreases when endogenous ABA levels are elevated, as observed with mannitol treatment. To further investigate this, we used the *aba deficient 1* (*aba1*), which has reduced levels of endogenous ABA (*39*), and therefore expected an increase of PD density. However, we found no significant difference in the punctate signal density of aniline blue staining between WT and *aba1* (fig. S1, E and F; mean punctate signal density in “WT” versus “*aba1*” = 1.413/μm versus 1.287/μm, *P* = 0.2012). Collectively, these results suggest that ABA-dependent PD density reduction is primarily induced under stress conditions when endogenous ABA levels are elevated.

### ABA decreases primary PD density in newly forming cell plates of brood cells

**Fig. 3.**
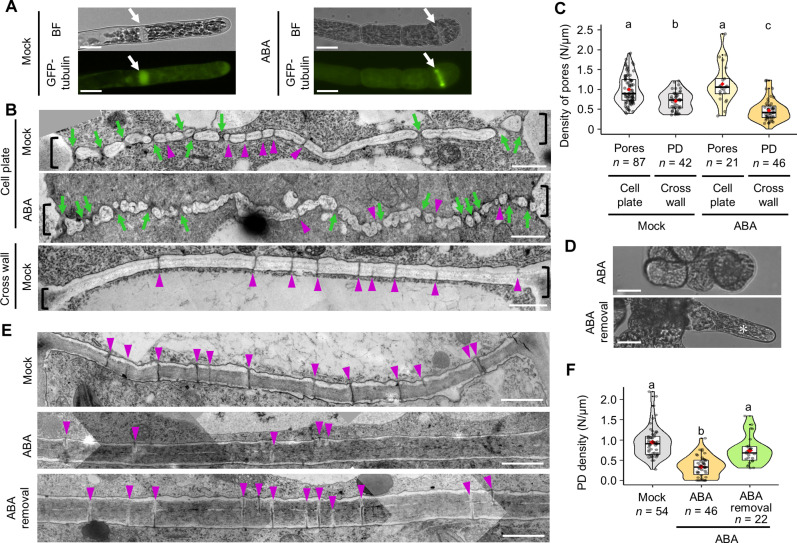
ABA reduces primary PD density in newly forming cell plates. (**A**) Protonemal cells of the GFP-tagged tubulin line (mock and ABA treatments). In both bright field (BF) and GFP fluorescence images, white arrows show the cell division plane. Scale bars, 15 μm. (**B**) Newly forming cell plates of the GFP-tubulin line during cell division (mock and ABA treatments) and mature cross walls (mock treatment). Magenta arrowheads indicate regular pores that are the same size as PD, and green arrows indicate pores larger than PD. Black brackets indicate the nexus of the side walls and the forming cell plates. Scale bars, 1 μm. (**C**) Quantification of the density of larger-sized pores and PD-sized pores in GFP-tubulin cell plates and cross walls under mock or ABA treatment. (**D**) Regeneration of chloronemal cells, a type of protonemal cell, from brood cells. Protonemal cells following treatment with ABA for 6 days (top) or 4 days followed by 2 days of growth on ABA-free medium (bottom). Asterisk indicates a newly emerged chloronemal cell. Scale bars, 15 μm. (**E**) TEM images of cross walls formed under mock or ABA treatment (4 days) and newly formed after ABA removal (4 days). Magenta arrowheads indicate PD-sized pores. Scale bars, 1 μm. (**F**) Quantification of PD density in cross walls formed under mock or ABA treatment (4 days) and newly formed after ABA removal (4 days). (C and F) The value of *n* indicates the number of different cross walls. One-way ANOVA followed by Tukey’s post hoc test, *P* < 0.01. Violin plots show numerical data. Box boundaries indicate the upper (75th percentile) and lower (25th percentile) quartiles, and the whiskers show 75th percentile + 1.5 IQR and 25th percentile – 1.5 IQR. Median and mean are indicated by a bold line and a red diamond, respectively.

Next, we investigated whether the lower PD density found in newly formed cross walls was due to a block in primary PD formation in newly forming cell plates. We observed primary PD formation in a *P. patens* line expressing green fluorescent protein (GFP)–tagged tubulin to aid in identifying phragmoplasts formed during late cytokinesis (e.g., anaphase to telophase; Fig. 3A). The newly forming cell plates (“cell plate” in Fig. 3B) were thinner than fully developed mature cross walls (“cross wall” in Fig. 3B) and did not completely adhere to the side walls (Fig. 3B; black brackets indicate the nexus between cell plate and side wall). We identified two types of pores in TEM observation of newly forming cell plates: larger-sized pores and regular pores that were the same size as PD (Fig. 3B, green arrows and magenta arrowheads, respectively). In contrast, we observed only PD-sized pores in mature cross walls (Fig. 3B).

We quantified the density of all larger-sized pores and PD-sized pores in TEM observation of cell plates and cross walls (Fig. 3C). In the mock treatment, cross walls have a lower pore density than cell plates (Fig. 3C; mean density in “Cell plate” versus “Cross wall” in mock treatment = 0.998/μm versus 0.7076/μm, *P* = 3.243 × 10^–7^). These data imply that some of the pores present in the cell plate became bona fide primary PD, while others disappeared and likely became embedded as a part of the cross wall. We then compared the density of all pores between the mock and ABA treatments. There was no significant difference in pore density in newly forming cell plates between the mock and ABA treatments (Fig. 3C; mean density of pores in “Cell plate” “Mock” versus “ABA” = 0.998/μm versus 1.14/μm, *P* = 0.2322). In contrast, the density of PD-sized pores in cross walls was lower under ABA treatment than under mock treatment (Fig. 3C; mean density of PD in “Cross wall” “Mock” versus “ABA” = 0.7076/μm versus 0.4724/μm, *P* = 5.088 × 10^–5^). Thus, the data suggest that ABA suppresses primary PD formation during the formation of new cross walls.

We next asked whether PD density could be recovered in newly formed cross walls after the removal of ABA. After transferring brood cells to an ABA-free environment, normal protonemal cells were regenerated by asymmetric cell division (Fig. 3D). TEM observation revealed that the PD density of cross walls newly formed after ABA removal was similar to that observed under mock treatment (Fig. 3, E and F; mean PD density in “ABA” versus “ABA removal” = 0.336/μm versus 0.736/μm, *P* = 4.797 × 10^–6^). Thus, our data suggest that the reduction in PD density is reversible and that cell-to-cell communication is dynamically regulated by modulating PD density according to the presence of ABA.

### ABA core module is crucial for the ABA-induced reduction in primary PD density

**Fig. 4.**
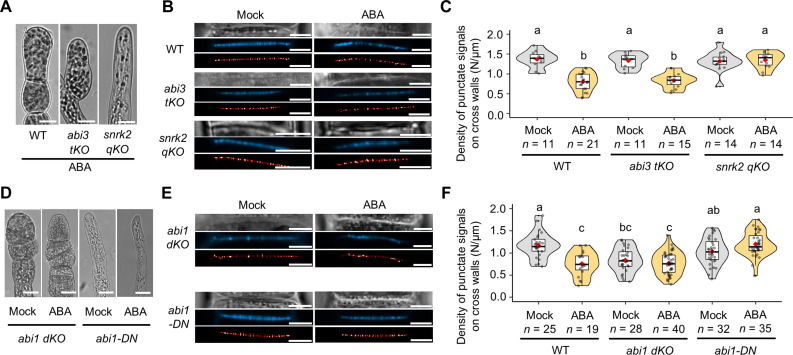
SnRK2 and ABI1 are essential for the ABA-induced decrease in PD density. (**A**) Brood cell formation after 7 days of ABA treatment in the WT, *abi3 tKO*, and *snrk2 qKO*. Scale bars, 15 μm. (**B**) Bright-field (top), aniline blue fluorochrome (middle), and SRRF image (bottom) of cross walls after 7 days of ABA treatment in the WT, *abi3 tKO*, and *snrk2 qKO*. Scale bars, 5 μm. (**C**) Quantification of the density of aniline blue fluorescent spots in cross walls formed in the WT, *abi3 tKO*, and *snrk2 qKO* after mock or ABA treatment. (**D**) Protonemal cells of *abi1 dKO* and *abi1-DN* after 4 days of treatment with 0.1% DMSO (mock) or ABA. Scale bars, 15 μm. (**E**) Bright-field (top), aniline blue fluorochrome (middle), and SRRF image (bottom) of cross walls after 7 days of ABA treatment in *abi1 dKO* and *abi1-DN* after 4 days of treatment with 0.1% DMSO (mock) or ABA. Scale bars, 5 μm. (**F**) Quantification of the density of aniline blue fluorescent spots in cross walls formed in the WT, *abi1 dKO*, and *abi1-DN* after mock or ABA treatment. (C and F) The value of *n* indicates the number of different cross walls. One-way ANOVA followed by Tukey’s post hoc test, *P* < 0.01. Violin plots show numerical data. Box boundaries indicate the upper (75th percentile) and lower (25th percentile) quartiles, and the whiskers show 75th percentile + 1.5 IQR and 25th percentile – 1.5 IQR. Median and mean are indicated by a bold line and a red diamond, respectively.

Recent studies have revealed the vital roles of the ABA core signaling pathway in the response to abiotic stress, which is well conserved among land plants (*29*, *31*, *32*). We previously demonstrated that short-term (<3 hours) ABA or salt stress treatment reduces PD permeability in *P. patens* protonemata and that the protein kinase, PpSnRK2, and the type 2C protein phosphatase, PpABI1, components of the ABA core module, are essential for the regulation of PD permeability by ABA; the B3-type transcription factor PpABI3 is partially involved in this regulation as a downstream factor (*26*). Although short-term ABA treatment does not alter PD density, we asked whether PpSnRK2, PpABI1, and PpABI3 might be involved in reducing PD density under long-term ABA treatment (>3 days). We first examined a *Ppabi3* triple knockout mutant (*abi3 tKO*) lacking the *PpABI3A*, *PpABI3B*, and *PpABI3C* genes (*40*). Morphology of *abi3 tKO* protonemal cells was similar to those of the WT before ABA treatment (fig. S2A), and ABA-treated *abi3 tKO* produced brood cells (Fig. 4A). In aniline blue staining, the punctate signal density in *abi3 tKO* was lower under ABA treatment than under mock treatment, to the same degree as in the ABA-treated WT (Fig. 4, B and C; mean punctate signal density in “WT ABA” versus “*abi3 tKO* ABA” = 0.8039/μm versus 0.8362/μm, *P* = 0.6459), suggesting that PpABI3 is not involved in the ABA-induced decrease in primary PD formation.

We then investigated the PD density in the *Ppsnrk2* quadruple mutant (*snrk2 qKO*), lacking the *PpSnRK2A*, *PpSnRK2B*, *PpSnRK2C*, and *PpSnRK2D* genes (*41*). Much as in the *abi3 tKO* mutant, the cell morphology of *snrk2 qKO* was comparable to that of the WT under normal conditions (fig. S2A). However, ABA-treated *snrk2 qKO* did not produce brood cells (Fig. 4A) and did not exhibit lower punctate signal density of aniline blue than mock-treated *snrk2 qKO* (Fig. 4, B and C; mean punctate signal density in *snrk2 qKO* “Mock” versus “ABA” = 1.318/μm versus 1.349/μm, *P* = 0.7228), highlighting the crucial role of PpSnRK2 in the formation of both brood cells and primary PD mediated by ABA. In a complementary approach, we inducibly overexpressed *PpABI3A* or *PpSnRK2A* (*26*). The overexpression of *PpABI3A* had little effect on cell shape (fig. S2B) and no significant effect on the punctate signal density of aniline blue (fig. S2, C and D; mean punctate signal density in “Mock” versus “β-est newly formed” = 1.083/μm versus 1.068/μm, *P* = 0.8062). In contrast, the overexpression of *PpSnRK2A* resulted in swollen cell morphology (fig. S2E); however, there was no significant difference in the punctate signal density of aniline blue between cells overexpressing *PpSnRK2A* and those that did not (fig. S2, F and G; mean punctate signal density in “Mock” versus “β-est newly formed” = 1.159/μm versus 1.087/μm, *P* = 0.2663).

We also investigated the aniline blue punctate signal density in mutants of *PpABI1*, another component of the ABA core module. Because PpABI1 is a negative regulator of ABA signaling (*42*), the *Ppabi1* double mutant (*abi1 dKO*) lacking the *PpABI1A* and *PpABI1B* genes produced brood cells even in the absence of ABA treatment (Fig. 4D). *abi1 dKO* showed a lower punctate signal density of aniline blue than the WT even without ABA treatment (mock), which was comparable to the punctate signal density observed in the ABA-treated WT (Fig. 4, E and F; mean punctate signal density in “WT ABA” versus “*abi1 dKO* mock” = 0.7553/μm versus 0.8366/μm, *P* = 0.3176). Moreover, the punctate signal density in mock-treated *abi1 dKO* was as low as that of ABA-treated *abi1 dKO* (Fig. 4F; mean punctate signal density in *abi1 dKO* “Mock” versus “ABA” =0.8366/μm versus 0.7671/μm, *P* = 0.2897). We next examined the punctate signal density of aniline blue in a line overexpressing the dominant negative form of *PpABI1A* (*abi1-DN*) (*26*); this line is ABA insensitive and did not show brood cell formation even under ABA treatment (Fig. 4D). As expected, *abi1-DN* plants displayed a punctate signal density similar to that of the mock-treated WT, even under ABA treatment (Fig. 4, E and F; mean punctate signal density in *abi1-DN* “Mock” versus “ABA” = 1.033/μm versus 1.200/μm, *P* = 0.0198). Thus, our data suggest that PpABI1 plays a pivotal role in the ABA-mediated regulation of PD density.

**Fig. 5.**
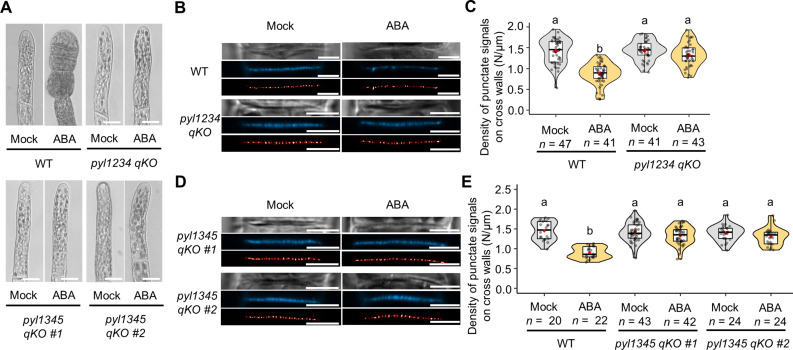
ABA receptors are essential for the ABA-induced decrease in PD density. (**A**) Protonemal cells of WT, *pyl1234 qKO*, and *pyl1345 qKOs* after 4 days of treatment with 0.1% DMSO (mock) or ABA. Scale bars, 15 μm. (**B**) Bright-field (top), aniline blue fluorochrome (middle), and SRRF image (bottom) of cross walls in WT and *pyl1234 qKO* after 4 days of treatment with 0.1% DMSO (mock) or ABA. Scale bars, 5 μm. (**C**) Quantification of the density of aniline blue fluorescent spots in cross walls formed in the WT and *pyl1234 qKO* after mock or ABA treatment. (**D**) Bright-field (top), aniline blue fluorochrome (middle), and SRRF image (bottom) of cross walls in *pyl1345 qKOs* after 4 days of treatment with 0.1% DMSO (mock) or ABA. Scale bars, 5 μm. (**E**) Quantification of the density of aniline blue fluorescent spots in cross walls formed in the WT and *pyl1345 qKOs* after mock or ABA treatment. (C and E) The value of *n* indicates the number of different cross walls. One-way ANOVA followed by Tukey’s post hoc test, *P* < 0.01. Violin plots show numerical data. Box boundaries indicate the upper (75th percentile) and lower (25th percentile) quartiles, and the whiskers show 75th percentile + 1.5 IQR and 25th percentile – 1.5 IQR. Median and mean are indicated by a bold line and a red diamond, respectively.

We next investigated whether the ABA receptor, the remaining ABA core component, is also involved in PD formation. We determined that the PYR/PYL/RCAR-type ABA receptor is well conserved between *P. patens* and *A. thaliana*, and molecular phylogenetic analysis revealed that five orthologous receptors are present in the moss (named *Pppyl1* and *Pp3c9_19760*, *Pppyl2* and *Pp3c7_26290*, *Pppyl3* and *Pp3c26_15240*, *Pppyl4* and *Pp3c13_7110*, and *Pppyl5* and *Pp3c3_660*) (fig. S3A). Assuming that the individual ABA receptors in the moss may function redundantly, as reported for many of the *A. thaliana* ABA receptors (*42*, *43*), we attempted to generate higher-order *pyl KO* mutants. Transcript data from the public databases (*44*) indicate that *PpPYL3* and *PpPYL4* are highly expressed in protonema (both chloronema and caulonema) (fig. S3B). Therefore, we aimed to create *PpPYL* quadruple or quintuple mutants, including *PpPYL3* and *PpPYL4*. While quintuple mutants were not achieved despite several attempts, we successfully generated three independent quadruple KO (qKO) mutants (*pyl1234 qKO*, *pyl1345 qKO #1*, and *pyl1345 qKO #2*) using CRISPR-Cas9 genome editing. The deletion of each *pyl* gene and the absence of their expression were confirmed by genomic polymerase chain reaction (PCR) and reverse transcription PCR for *pyl1234 qKO* (fig. S3, C and D), while the mutations in *pyl1345 qKO #1* and *pyl1345 qKO #2* were confirmed by nucleotide sequencing (fig. S3E). Phenotypic analysis revealed that all three *pyl qKO* mutants were insensitive to exogenous ABA and did not form brood cells after 4 days of ABA treatment (Fig. 5A), suggesting that these proteins function as bona fide ABA receptors in *P. patens*. These mutants did not show any alteration in the punctate signal density of aniline blue staining, even under 4 days of ABA treatment (Fig. 5, B to E; mean punctate signal density in *pyl1234 qKO* “Mock” versus “ABA” = 1.446/μm versus 1.321/μm, *P* = 0.035; *pyl1345 qKO #1* “Mock versus ABA” = 1.409/μm versus 1.336/μm, *P* = 0.156, *pyl1345 qKO #2* “Mock versus ABA” = 1.390/μm versus 1.300/μm, *P* = 0.144). Notably, neither *pyl1234 qKO* mutants (retaining *PYL5*) nor *pyl1345 qKO* mutants (retaining *PYL2*) exhibited ABA-dependent reductions in punctate signal density. Together, our data suggest that the ABA core components—PYR/PYL/RCAR-type ABA receptor, SnRK2 kinase, and ABI1 regulatory phosphatase—are crucial for the ABA-mediated reduction in primary PD density.

### ABI5 works as a downstream factor in the regulation of PD density by ABA

**Fig. 6.**
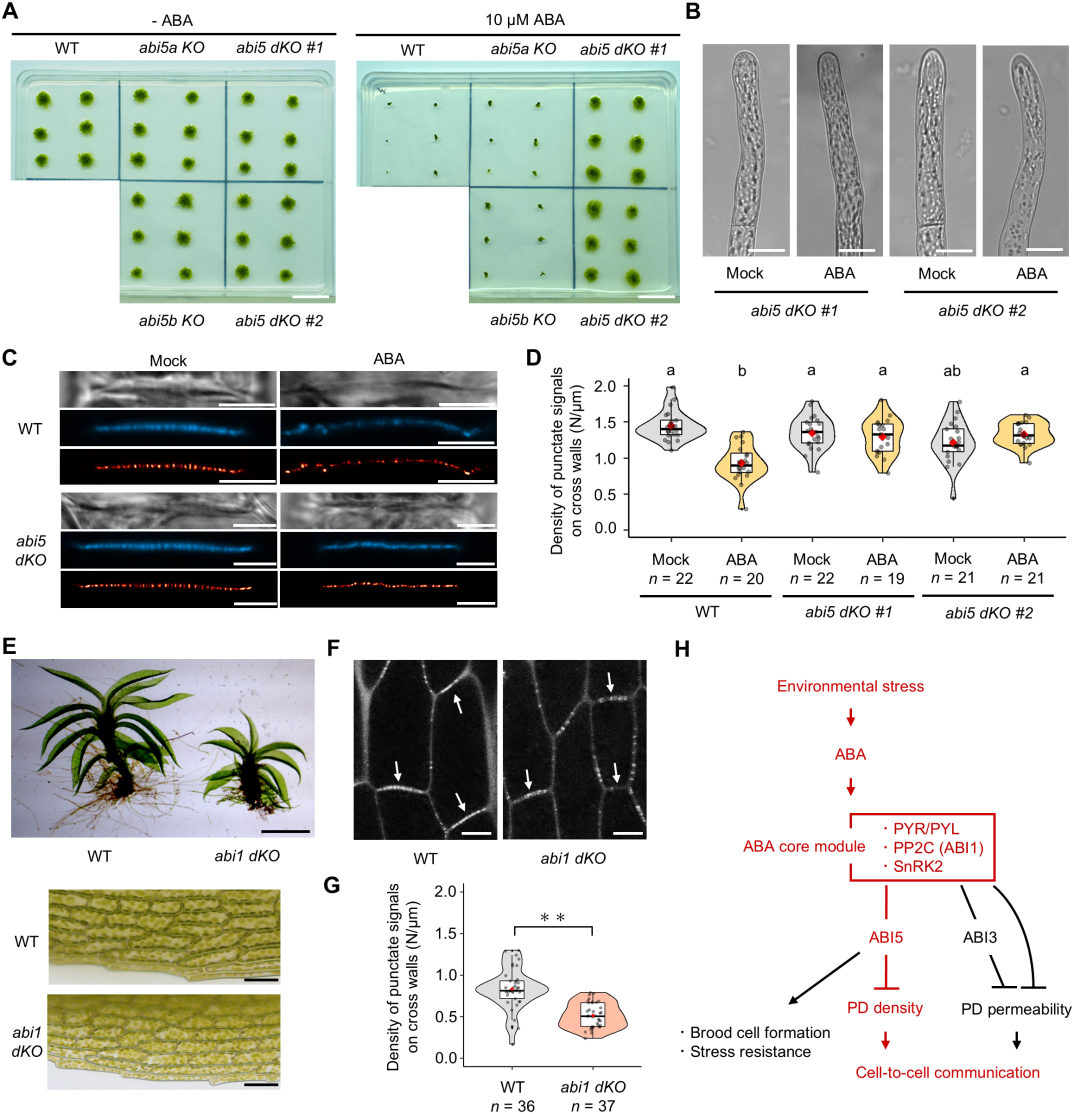
ABI5 works as a downstream factor in the regulation of PD density by ABA. (**A**) Colonies of *abi5* single KO and double KO (*dKO*) mutants after 3 weeks with or without ABA treatment. (**B**) Protonemal cells of *abi5 dKO* after 4 days of 0.1% DMSO (mock) or ABA treatment. Scale bars, 15 μm. (**C**) Bright-field (top), aniline blue fluorochrome (middle), and SRRF image (bottom) of cross walls in *abi5 dKO* after 4 days of 0.1% DMSO (mock) or ABA treatment. Scale bars, 5 μm. (**D**) Quantification of the density of aniline blue fluorescent spots in cross walls formed in *abi5 dKO* after mock or ABA treatment. (**E**) Leafy shoots, gametophores (top), and phyllid cells (bottom) of the WT and *abi1 dKO*. Scale bars, 1 mm (top) and 30 μm (bottom). (**F**) Aniline blue fluorochrome of a gametophore leaf in the WT and *abi1 dKO*. White arrows indicate the short axis of cross walls. Scale bars, 10 μm. (**G**) Quantification of the density of aniline blue fluorescent spots in short cross walls in the WT and *abi1 dKO* (*P* < 0.01, Welch’s *t* test). (**H**) A model of the role of the ABA core signaling module in environmental stress tolerance, cell fate decision, and cell-to-cell communication by regulating PD density and permeability. The pathways elucidated in this study are shown in red. (D and G) The value of *n* indicates the number of different cross walls. One-way ANOVA followed by Tukey’s post hoc test, *P* < 0.01. Violin plots show numerical data. Box boundaries indicate the upper (75th percentile) and lower (25th percentile) quartiles, and the whiskers show 75th percentile + 1.5 IQR and 25th percentile – 1.5 IQR. Median and mean are indicated by a bold line and a red diamond, respectively.

PpABI3, one of the downstream transcription factors of the ABA core module, was not critical for the regulation of PD density (Fig. 4, B and C). We therefore looked for any other downstream factors that are important for the regulation of PD density. ABI5 is a leucine zipper–type transcription factor and one of the key substrates of SnRK2 in ABA responses in *A. thaliana* (*45*, *46*). In *P. patens*, a portion of the peptide fragment of ABI5 was identified as a direct phosphorylation target of SnRK2 (*41*). Thus, ABI5 might be another good candidate for the regulation of PD density. Basic Local Alignment Search Tool (BLAST) search and phylogenetic analysis identified three *ABI5* orthologs (*Pp3c20_7230*, *Pp3c20_7290*, and *Pp3c23_19420*) in the *P. patens* genome, named *PpABI5A*, *PpABI5B*, and *PpABI5C*, respectively (fig. S4A). The protein-coding sequences of *PpABI5A* and *PpABI5B* were identical, whereas the 5′ and 3′ untranslated regions were divergent, implying that these genes arose from a recent duplication. Northern blot analysis indicated that the transcription of *PpABI5A*, *5B,* and *5C* is induced by ABA, with a more pronounced ABA response observed in *PpABI5A* and *PpABI5B* compared to *PpABI5C* (fig. S4B). Consequently, we focused our functional analyses on *PpABI5A* and *PpABI5B*. In addition to the direct phosphorylation of ABI5 by SnRK2 (*41*), we confirmed a physical interaction between PpABI5A (and PpABI5B) and PpSnRK2 by yeast two-hybrid assay (fig. S4C). An in vivo transactivation assay further revealed that PpABI5A and PpABI5B transcriptionally activate the ABA-responsive promoter of the *PpLEA1* gene in an ABA- and SnRK2-dependent manner (fig. S4D), confirming the functional importance of PpABI5A and PpABI5B as a positive transcription factor of canonical ABA signaling. Single KO mutants of *PpABI5A* or *PpABI5B* showed an ABA response comparable with that of the WT, exhibiting severe growth retardation under 10 μM ABA (Fig. 6A). Because these results implied that PpABI5A and PpABI5B function redundantly, we further generated double KO mutants of *PpABI5A* and *PpABI5B* (fig. S4E). The resultant *Ppabi5ab* double mutants (*abi5 dKO*) showed much lower sensitivity to ABA than the WT or single mutants (Fig. 6A); loss of ABA sensitivity was confirmed by observing that the protonemal cells of the mutants did not form brood cells under ABA treatment (Fig. 6B). The cross walls of *abi5 dKO* showed a similar punctate signal density of aniline blue after 4 days of ABA treatment to those under mock treatment (Fig. 6, C and D; mean punctate signal density in *abi5 dKO #1* “Mock” versus “ABA” = 1.401/μm versus 1.295/μm, *P* = 0.260; *abi5 dKO #2* “Mock” versus “ABA” = 1.212/μm versus 1.361/μm, *P* = 0.077). These results reveal that PpABI5A and PpABI5B constitute crucial downstream factors and play a pivotal role in the ABA-mediated regulation of PD density.

### ABA signaling potentially regulates primary PD density across different cell types

We observed that brood cell formation and PD density reduction were tightly coupled, suggesting that regulation of PD density by ABA signaling might be cell fate or cell morphology specific. To verify this, we investigated PD density in phyllid (leaf) cells of leafy shoots, gametophores, of the *abi1 dKO* mutant, in which the ABA signaling pathway is constantly activated (*47*). Brood cells with rounded cell morphology were formed in protonemal tissues even in the absence of ABA treatment (Fig. 4D). Notably, however, the overall morphology of the gametophore in the mutant was similar to that in the WT, although the mutant gametophores were stunted; the phyllid cells did not show a rounded cell morphology, instead maintaining a similar rectangular shape as seen in the WT (Fig. 6E). Using a confocal microscope, we distinguished the short-axis and long-axis sides of the cross walls in phyllid cells. To minimize potential variations in PD density between these sides, we measured the punctate signal density on the short-axis side of the cross walls using aniline blue staining (Fig. 6F, white arrows). Phyllid cells from the *abi1 dKO* mutant had a significantly lower punctate signal density than those from the WT (Fig. 6, F and G; mean punctate signal density in “WT” versus “*abi1 dKO*” = 0.831/μm versus 0.515/μm, *P* = 1.445 × 10^–8^). Thus, our results indicate that the regulation of PD density by ABA also occurs in cells other than brood cells. Recently, the presence of secondary PD was reported in the phyllid of gametophores in *P. patens* (*48*); therefore, we cannot entirely rule out the possibility that ABA influences secondary PD density in phyllid cells. However, the same study also reported a low occurrence of branched PD (less than 5%) in the phyllid development (*48*). Thus, we speculate that ABA primarily affects primary PD formation, consistent with our observations in protonema. Together, our results suggest that, regardless of changes in cell morphology, the ABA signaling pathway regulates primary PD density across different cell types (Fig. 6H).

## DISCUSSION

To date, there have been few reports on the regulation of primary PD density in Phragmoplastophyta species. Cytokinins and salicylic acid promote PD formation in angiosperms (*22*–*24*), but their functions are related to secondary PD formation. Furthermore, because primary PD are critically important for multicellularity, and their absence is thought to be lethal, mutants lacking primary PD have yet to be obtained. It is also difficult to observe changes in the number and density of primary PD during cytokinesis in angiosperms owing to their more complex multicellularity compared with the simple filamentous tissue of moss protonema. Recent studies in angiosperms have revealed that ABA and the ABA signaling components play notable roles in suppressing PD permeability via callose deposition at PD; this results in the restriction of cell-to-cell movement of viruses, water and auxin fluxes, and the seasonal growth–promoting signal FLOWERING LOCUS T 1, which affects plant immunity, lateral root branching, and bud dormancy, respectively (*27*, *28*, *49*). However, it remains to be seen whether ABA or its signaling components regulate primary PD density in angiosperms.

Currently, primary PD are hypothesized to form when the ER network becomes trapped within the expanding cell plate during cell division, creating pores that develop into PD (*12*). This hypothesis, based on electron microscopy observations, underpins our focus on ER distribution in this study. To investigate the effect of ABA on ER distribution, we performed live-cell imaging using an ER marker, mCherry–His-Asp-Glu-Leu (HDEL), given the challenges of visualizing ER strands trapped in the cell plate during PD formation by TEM (Fig. 3B). Observations were conducted 4 days after ABA treatment, a time point at which PD density reduction under ABA treatment was evident. In mock-treated cells, tubular ER was distributed throughout the cytoplasm (fig. S5A, white arrowheads). In contrast, ABA-treated cells displayed less tubulated ER throughout the cytoplasm (fig. S5A, white arrows), suggesting that ABA influences global ER organization. When we examined ER localization during cell division, mock-treated cells exhibited a clear ER distribution at the cell division plane, with ER strands extending perpendicularly to the division plane (fig. S5B, white arrowheads). However, ABA-treated cells exhibited less tubulated ER near the cell division plane (fig. S5B, white arrows). These findings indicate that ABA disrupts ER morphology or distribution at the cell division plane, potentially influencing primary PD formation. In addition, ABA may inhibit primary PD formation by adversely affecting cell plate formation. ABA could disrupt vesicle trafficking, membrane fusion, or other cellular processes essential for cell plate assembly (*34*, *50*, *51*), thereby altering PD formation. While our study provides evidence for ABA’s role in regulating ER morphology and PD formation, the precise molecular mechanisms remain to be elucidated. Future experiments will aim to determine how less tubulated ER near the cell division plane influences the establishment of primary PD and to identify additional downstream factors in the ABA signaling pathway contributing to this regulation. For instance, investigating factors downstream of ABI5 may shed light on the direct regulation of primary PD formation.

Given that ABA is a universal phytohormone in land plants and that the ABA core signaling module and the downstream transcription factors were first established in bryophytes (*29*, *31*, *32*), we speculate―based on our findings and previous studies on the ABA-mediated regulation of PD permeability (*25*, *26*)―that the coordinated activation of stress tolerance genes and the regulation of PD density and/or permeability by the ABA signaling pathway in response to abiotic stresses may represent a conserved and widespread mechanism in land plants (Fig. 6H).

## MATERIALS AND METHODS

### Plant materials and growth conditions

The moss *P. patens* (Hedw., previously *Physcomitrella patens*) Mitten was used in this study as the WT. The lines *ProEF1*α*:D2* and *ProEF1α:D2* in *ppaba1* background (*aba1*), *Ppsnrk2qko* in the *ProEF1α:D2* background (*snrk2 qKO*), *Ppabi3tko* in the *ProEF1α:D2* background (*abi3 tKO*), *PpSnRK2AiOX* in the *ProEF1α:D2* background (*SnRK2iox*), and *PpABI3AiOX* in the *ProEF1α:D2* background (*ABI3iox*) were described previously (*25*, *52*). *PpABI1A^G333D^OX*/ProEF1α*:D2*, which was used as an overexpression line of the dominant negative form of *ABI1*, was described previously (*26*). The GFP-tubulin line was used as a tubulin marker (*53*). All plants were subcultured on a BCDAT medium solidified with 0.8% (w/v) agar (Nacalai Tesque) under continuous white light (30 μmol/m^2^ per second) at 25°C (*54*).

### Treatment and observation of protonemata

Protonemata tissues were prepared in glass-bottom dishes (four-well glass-bottom chamber, IWAKI) to enable detailed observations of cross-wall positions under an optical microscope. BCDATG [BCDAT with 0.5% (w/v) glucose] solidified with 0.8% (w/v) agar was used as culture medium. After the moss was transplanted onto the bottom of the chamber, the chamber was turned upside down and incubated under continuous red light (20 μmol/m^2^ per second) for 5 to 7 days. The position of the cross walls was checked under an inverted microscope (ECLIPSE Ti-E, Nikon) before and at 4 days after the administration of 0.1% (v/v) dimethyl sulfoxide (DMSO) (mock treatment) or 50 μM ABA.

To remove the ABA, the glass-bottom chamber treated with ABA was submerged in 100 ml of liquid BCDATG medium for 24 hours. The liquid medium was discarded, and the protonemata were observed. The culture was transferred to red light (20 μmol/m^2^ per second) and incubated for 4 days.

For mannitol treatment of the WT and ABA treatment of *snrk2 qKO* and *abi3 tKO*, mosses were grown in liquid BCDAT medium in 24-well plates for 5 days with continuous white light. Mannitol was applied at final concentrations of 0.1 and 0.3 M, and ABA was applied at a final concentration of 50 μM, followed by incubation for 7 days (mannitol) or 4 days (ABA).

### Phylogenetic analysis of the PYR/PYL/RCAR ABA receptor and the group A basic leucine zipper–type transcription factor ABI5

To reconstruct the PYR/PYL/RCAR phylogenetic tree, full-length protein sequences were aligned using MUSCLE (*55*) implemented in MEGA11 software (https://megasoftware.net/) with default parameters. A rooted maximum-likelihood phylogenetic tree was reconstructed using LG as the substitution model. Numbers on branches denote bootstrap support based on 100 repetitions.

To reconstruct the ABI5 phylogenetic tree, full-length protein sequences were aligned using MUSCLE (*55*) implemented in the Geneious Prime software package (Biomatters; www.geneious.com) with default parameters. A rooted maximum-likelihood phylogenetic tree was reconstructed using the PhyML program (*56*) implemented in the Geneious Prime software, with LG as the substitution model. Numbers on branches denote bootstrap support based on 100 repetitions.

### Establishment of *abi5 dKO* lines

To create the targeting constructs for *PpABI5A* (*Pp3c20_7230*) and *PpABI5B* (*Pp3c20_7290*), regions spanning approximately 2 kb upstream and downstream of the open reading frames [2.8 kb (5′) and 3.3 kb (3′) for *PpABI5A* and 2.5 kb (5′) and 2.5 kb (3′) for *PpABI5B*] were amplified by PCR using KOD -Plus- Neo (TOYOBO). The amplified fragments were verified by sequencing and cloned into Lox-CaMV35S::NPTII::NOS-Lox (*57*) (*PpABI5A*) using Sfi I (5′ fragment) and Asc I (3′ fragment) and Lox-CaMV35S::HPT::NOS-Lox (*41*) (*PpABI5B*) using Not I–Sal I (5′ fragment) and Nsi I–Asc I (3′ fragment), respectively.

The *P. patens* WT was used to establish *Ppabi5a* or *Ppabi5b* single disruptants by transforming the *PpABI5A* or *PpABI5B* targeting construct into *P. patens* via polyethylene glycol (PEG)–mediated protoplast transformation (*54*), with geneticin (20 μg/ml) or hygromycin (30 μg/ml) for the screening of *Ppabi5a KO* or *Ppabi5b KO* plants, respectively. The resulting *Ppabi5a KO* plant was used for generating the *Ppabi5a/b* double disruptant (*abi5 dKO*), with hygromycin (25 μg/ml) for screening. Because the sequence similarity between *PpABI5A* and *PpABI5B* is quite high, the double disruption of *PpABI5A/B* was confirmed using a single genomic PCR reaction with a common primer set (table S1).

### Establishment of *pyl qKO* lines

The Cas9 and single guide RNA (sgRNA) plasmids (*58*) and the associated gRNA primers were used for genome editing of *PpPYL* genes (table S1). The respective 19–base pair oligonucleotides were subcloned into pUC18 downstream of the *P. patens* U6 promoter. These targeting constructs were mixed with the Cas9-expression cassette driven by the actin promoter and BNRF plasmid for resistance to G418 (5 μg each) and used for PEG-mediated protoplast transformation (*54*) of protonemata from the *P. patens* WT. Colonies were selected on medium containing G418 and then screened on medium containing 10 μM ABA. In *pyl1234 qKO*, a large unknown deletion in each gene was confirmed by genomic PCR using the primer sets shown in table S1.

### Establishment of pGX8-mCherry-HDEL marker lines

To generate pGX8-mCherry-HDEL inducible construct, sequences including N-terminal signal peptide, mCherry tag and a C-terminal–attached ER retention signal, and HDEL were PCR amplified using PrimeSTAR Max DNA polymerase (Takara) from pGWB701-UBQ10-mCherry-HDEL plasmid provided by T. Junpei at Hokkaido University. The amplified sequence was cloned into pENTR1A (Thermo Fisher Scientific) and sequenced, before being subcloned into a modified pGX8 with zeocin antibiotic cassette (*59*) using reactions between attL and attR sites to generate pGX8-mCherry-HDEL: Zeocin. The list of primers used for this plasmid construction is listed in table S1.

To generate transgenic moss, protoplasts of WT moss were prepared and transformed with 15 μg of plasmid DNA. The PEG-mediated transformation was performed as described previously (*54*). The final transgenic lines were selected twice on BCDAT agar medium containing the appropriate antibiotics.

### Northern blot of PpABI5

Total RNA was extracted from protonemal tissues using an RNeasy Plant Mini Kit (QIAGEN). Approximately 10 μg of total RNA was separated in a denaturing formaldehyde agarose gel and transferred onto a nylon membrane. DNA probes corresponding to the first exon of PpABI5A/B or the second exon of PpABI5C were prepared by genomic PCR using the primer set shown in table S1. Hybridization with ^32^P-labeled DNA probes was carried out as described previously (*57*). A BAS-2500 imaging analyzer (Fujifilm) was used for visualization of the blot.

### Yeast two-hybrid assay between PpABI5A and PpSnRK2s

A cDNA harboring the open reading frame of *PpABI5A* was cloned into the multicloning site of pGADT7 (Clontech) in-frame with the GAL4 activation domain (AD) sequence. The open reading frame of *PpSnRK2A/B/C* was cloned into the multicloning site of pGBKT7 (Clontech) in-frame with the GAL4 binding domain (BD) sequence. Because *PpSnRK2D* cloned into pGBKT7 produced PpSnRK2D with strong autoactivation activity, it was not used for the assay. Diploid yeast cells harboring the indicated BD or AD constructs, or empty vector as control, were used for yeast two-hybrid plate assays with the reporter genes *GAL2-ADE2* and *GAL1-HIS3*. Colonies were grown for 1 week on synthetic dropout medium lacking leucine and tryptophan (SD – LW) or lacking leucine, tryptophan, histidine, and adenine (SD – LWHA).

### Transactivation of the *PpLEA1* promoter by PpABI5

A transient assay in *P. patens* protonemata was performed as previously described (*54*) to assess the requirement of SnRK2 for the transactivation ability of (effects of phosphorylation of) *PpABI5A/B*. The *PpLEA1* gene promoter fused to the beta-glucuronidase (GUS) gene (*PpLEA1-GUS*) was used as a reporter construct, and *PpABI5A/B* coding sequences driven by a rice (*Oryza sativa*) actin promoter (*Act-PpABI5*) were used as an effector construct. *P. patens* protonemata from the WT, *abi5 dKO*, and *snrk2 qKO* were bombarded with *PpLEA1-GUS* and *Ubi-LUC*, with or without the corresponding effector construct, using a PDS-1000/He device (Bio-Rad; www.bio-rad.com). The bombarded protonemata were cultured with or without 10 μM ABA for 24 hours. Proteins extracted from the protonemata were subjected to GUS and luciferase (LUC) assays (*38*). The extraction buffer is composed of 100 mM sodium phosphate (pH 7), 10 mM dithiothreitol, 0.02% (w/v) leupeptin, and 20% glycerol. Promoter activities were evaluated using the GUS/LUC ratio.

### Transmission electron microscopy

The positional information about the cross walls in each protonemal filament was recorded by inverted microscopy and excised from the agar media in a glass-bottom dish with moss for fixation and embedding for TEM observation. After fixation, during the trimming process of the embedded samples, the positional information about the same filament was checked again with a stereo microscope to distinguish the cross walls in a section to illustrate which cross walls were preformed or newly formed ones, and then the samples were cut into thin sections (thickness, 80 nm). Protonemata tissues were fixed in 3% (w/v) glutaraldehyde (Nisshin EM) and 1% (w/v) formaldehyde (freshly prepared from paraformaldehyde; TAAB) in 0.05 M sodium phosphate buffer (pH 7) for 2 hours at room temperature. After being rinsed in 0.1 M sodium phosphate buffer, the material was postfixed with 1% (w/v) osmium tetroxide (Nisshin EM) in 0.1 M sodium phosphate buffer (pH 6.8) overnight at 4°C, dehydrated through an ethanol series, and embedded in Spurr resin [mixing ERL4221, DER736, and NSA (Polysciences)]. Sections were sequentially stained with EM Stainer (Nisshin EM) for 30 min, stained with lead stain solution (Sigma-Aldrich Japan) for 8 min, and examined under an H-7650 transmission electron microscope (Hitachi High-Tech).

### Aniline blue staining

Aniline blue (Bio Supplies) was dissolved in sterile water to 1 mg/ml and stored as a stock solution at 4°C in the dark. When glass-bottom chambers were used, fresh solutions were prepared by diluting the solution to 330 μg/ml in 100 mM phosphate buffer (pH 8.5) and used to treat samples for 1 day before observation at a final concentration of 33 μg/ml. Moss plantlets grown in liquid medium were picked out, placed in fresh solution diluted to 33 μg/ml in 100 mM phosphate buffer (pH 8.5), and incubated at room temperature for 30 min. Gametophores were picked out, placed in fresh solution, and incubated for 6 hours at room temperature. Aniline blue fluorescence was detected under a Zeiss LSM 980 confocal microscope equipped with a Plan-Apochromat 63×/1.40 oil DIC M27 objective. The pinhole size of the confocal microscope was set to 0.8 Airy units, which corresponds to approximately 1.33 μm in optical section thickness. A 405-nm laser with 0.2% power intensity was used to excite aniline blue. The detection range was 410 to 605 nm. Fluorescence spot density was measured from super-resolution radial fluctuation (SRRF) images. NanoJ-SRRF (*60*), a plug-in for ImageJ (Fiji), was used to obtain one SRRF image from 10 consecutive images. Parameters for SRRF analysis were set to 8 for ring radius, 10 for radiality magnification, 8 for axes in ring, and Temporal Radiality Pairwise Product Mean for advanced setting.

### Quantification and statistical analysis

SRRF images were processed by ImageJ’s Filter and Threshold for binary images. The number of spots was automatically counted using the ImageJ application, Analyze particles. Spot density was calculated on the basis of the length of the area where aniline blue fluorescence was observed in the cross walls. Statistical analyses were performed using R version 4.0.2. For the quantitative graphs of PD density, the violin plot and scatter plots show the entire dataset, while the whiskers in the box plots show the maximum and minimum values [first quartile – 1.5 interquartile range (IQR) and third quartile + 1.5 IQR, respectively] excluding outliers. For multiple sample comparison, one-way analysis of variance (ANOVA) with Tukey post hoc test was performed. For two-sample comparison, a Welch’s or Student’s *t* test was performed. Graphs were generated using the R ggplot2 package, BoxPlotR, or Microsoft Excel. The value of *n* indicates the number of different cross walls. Statistical significance is defined in the figure legends. Statistical significance was set at *P* < 0.01.
