# Supplementary material for: Abscisic acid signaling regulates primary plasmodesmata density for plant cell-to-cell communication
Source: Sci Adv. 2025 May 7;11(19):eadr8298. doi: 10.1126/sciadv.adr8298 (PMC12057679; doi:10.1126/sciadv.adr8298)
Supplement: Supplementary file 1 — Figs. S1 to S5 Table S1 [file sciadv.adr8298_sm.pdf]

Science Advances
AAAS

Supplementary Materials for

Abscisic acid signaling regulates primary plasmodesmata density for plant cell-to-cell communication

Chiyo Jinno *et al.*

Corresponding author: Yoichi Sakata, sakata@nodai.ac.jp; Daisuke Takezawa, takezawa@mail.saitama-u.ac.jp; Tomomichi Fujita, tfujita@sci.hokudai.ac.jp

Sci. Adv. **11**, eadr8298 (2025)
DOI: 10.1126/sciadv.adr8298

This PDF file includes:

Figs. S1 to S5
Table S1

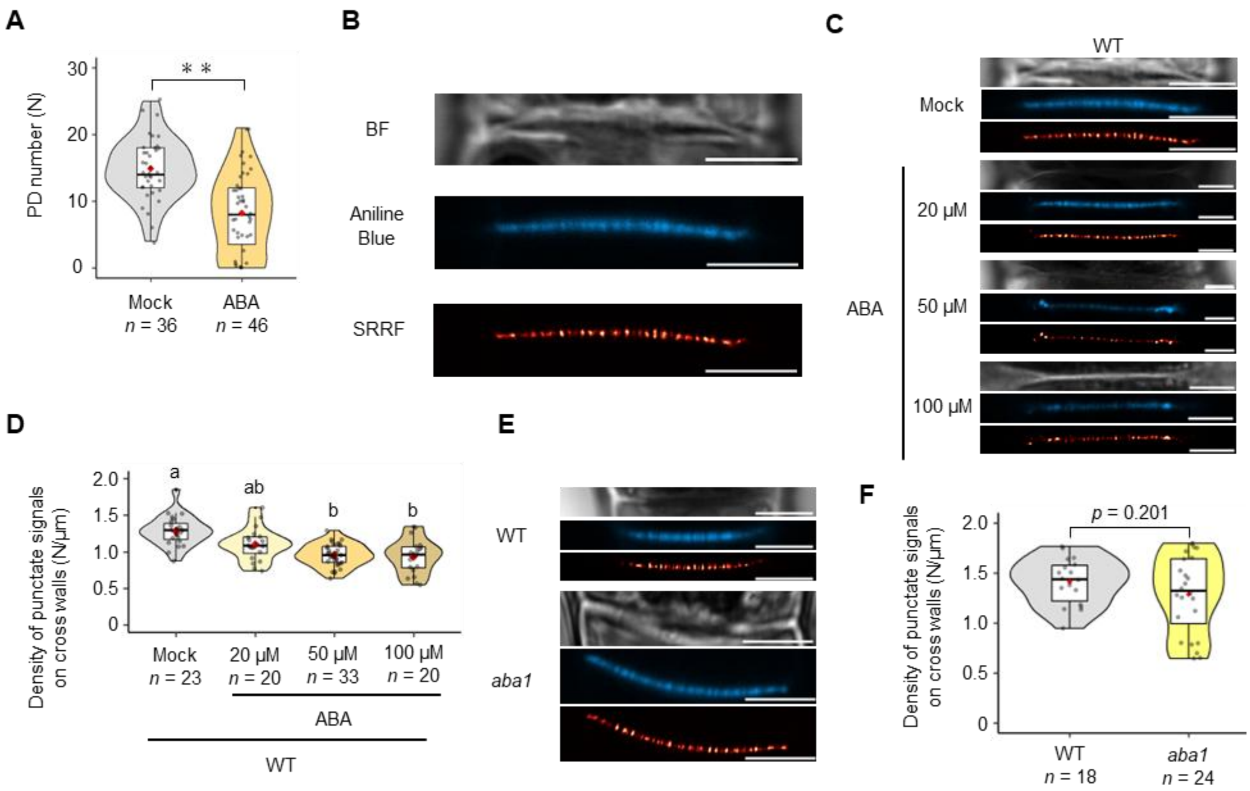

Fig. S1.

ABA affects PD number and density. (A) Quantification of PD number on the sections of cross walls in the 7 d of mock or ABA treatment WT using TEM ($p < 0.01$, Welch's t-test). Note that the overall length of cross walls varies depending on the sectioning location. **(B)** Super-resolution radial fluctuations (SRRF) image analysis of a WT cross wall. Top to bottom: bright-field image (BF), aniline blue fluorochrome image, and image obtained using the ImageJ plug-in nanoJ-SRRF. The parameters used in the SRRF analysis are described in Method. Bars, 5 µm. **(C)** Bright field (top), aniline blue fluorochrome (middle) and SRRF image (bottom) of wild-type cross walls under mock treatment or ABA treatment with different concentrations. Bars, 5 µm. **(D)** Quantification of the density of aniline blue fluorescent spots in cross walls formed under ABA treatment with different concentrations. **(E)** Bright field (top), aniline blue fluorochrome (middle) and SRRF image (bottom) of cross walls in wild-type and *aba1*. Bars, 5 µm. **(F)** Quantification of the density of aniline blue fluorescent spots in wild-type and *aba1* cross walls. **(D, F)** The value of n indicates the number of different cross walls. One-way ANOVA followed by Tukey's HSD test, $p < 0.01$. Violin plot shows numerical data. Box boundaries indicate the upper (75th percentile) and lower (25th percentile) quartiles, and the whiskers show 75th percentile-1.5 IQR and 25th percentile+1.5 IQR. Median and mean are indicated by a bold line and a red diamond, respectively.

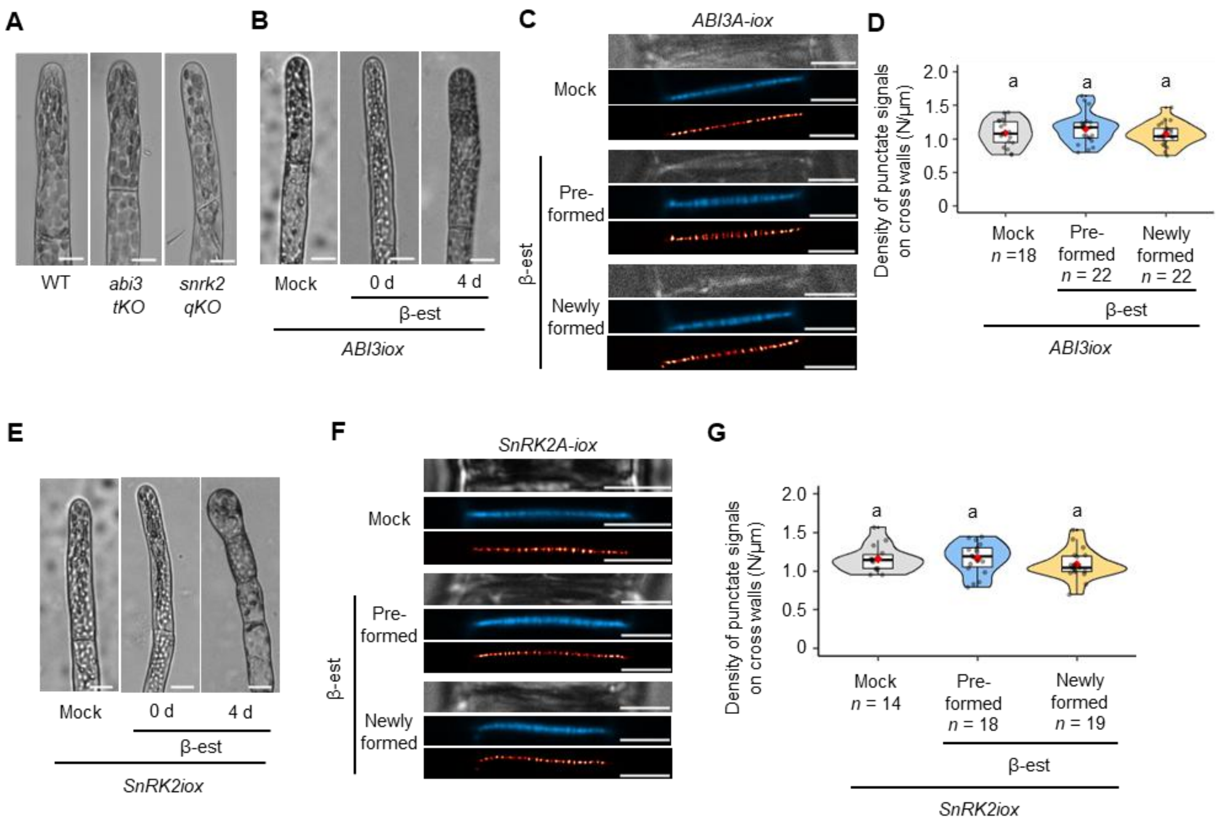

Fig. S2.

Overexpressing *ABI3* and *SnRK2* does not affect PD density. (A) Protonemal cells of WT, *abi3 tKO*, and *snrk2 qKO* before ABA application. Scale bars, 10 µm. **(B)** Protonemal cells of *ABI3A*-inducible overexpression line (*ABI3iox*) under mock (4 days) and β-estradiol treatment. Scale bars, 10 µm. **(C)** Bright field (top), aniline blue fluorochrome (middle) and SRRF image (bottom) of *ABI3iox* cross walls under mock treatment and cross walls pre-formed before and newly formed after β-estradiol treatment. Bars, 5 µm. **(D)** Quantification of the density of aniline blue fluorescent spots in the *ABI3iox* cross walls formed under mock treatment or pre-formed before and newly formed after the induction of overexpression by β-estradiol. **(E)** Protonemal cells of *Snrk2A*-inducible overexpression line (*SnRK2iox*) under mock (4 days) and β-estradiol treatment. Scale bars, 10 µm. **(F)** Bright field (top), aniline blue fluorochrome (middle) and SRRF image (bottom) of *SnRK2iox* cross walls under mock treatment and cross walls pre-formed before and newly formed after β-estradiol treatment. Bars, 5 µm. **(G)** Quantification of the density of aniline blue fluorescent spots in the *SnRK2iox* cross walls formed under mock treatment or pre-formed before and newly formed after β-estradiol application. **(D, G)** The value of n indicates the number of different cross walls. one-way ANOVA followed by Tukey's HSD test, $p < 0.01$. Violin plots show numerical data. Box boundaries indicate the upper (75th percentile) and lower (25th percentile) quartiles, and the whiskers show 75th percentile+1.5 IQR and 25th percentile-1.5 IQR. Median and mean are indicated by a bold line and a red diamond, respectively.

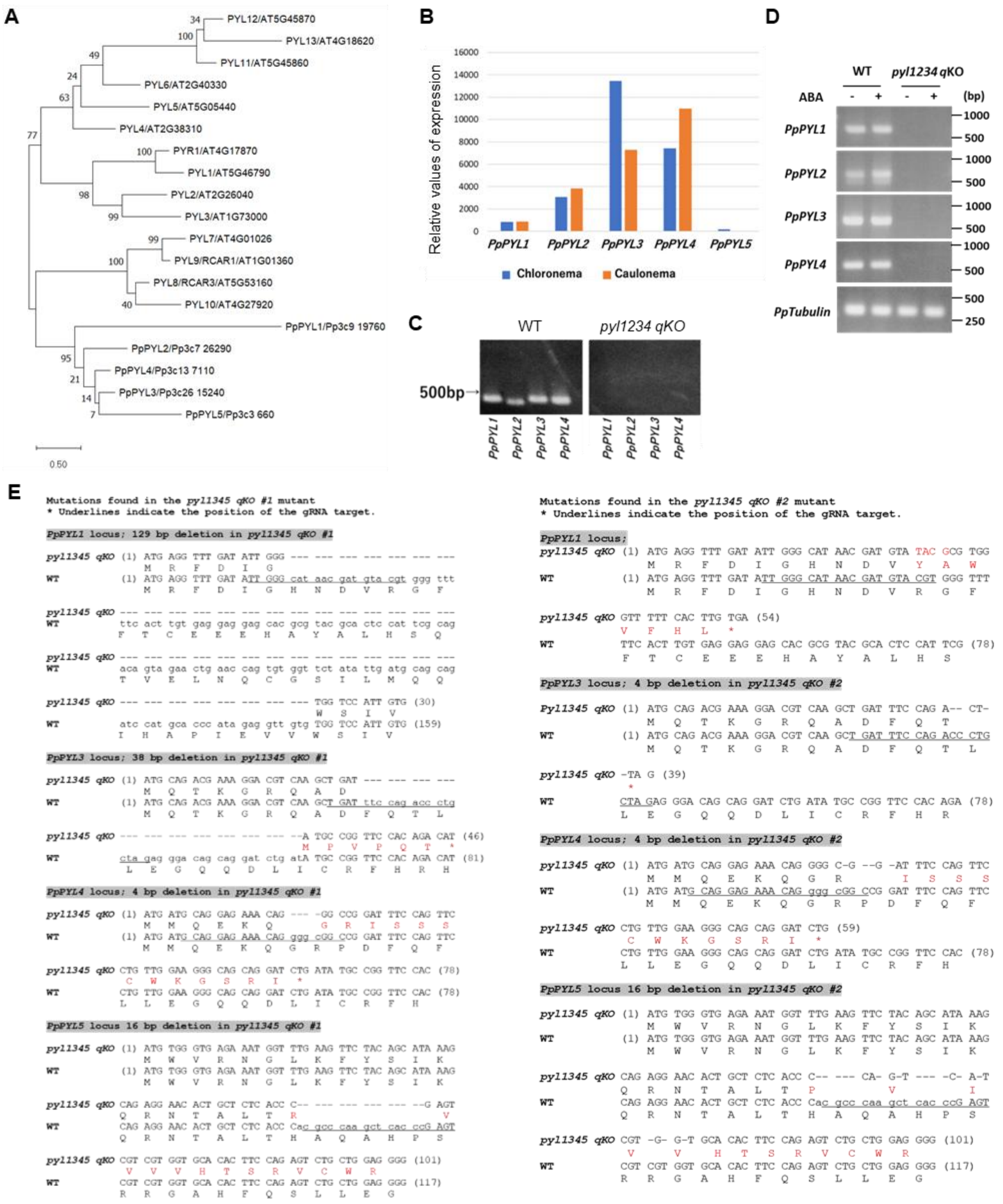

Fig. S3.

Knockout of *PpPYLs* in *P. patens*. (A) Phylogenetic tree of PYL receptors in *P. patens* and *A. thaliana*. A non-rooted maximum-likelihood phylogenetic tree was reconstructed using LG as the substitution model. Numbers on branches denote bootstrap support based on 100 repetitions. Bar, number of amino acid changes per branch length. **(B)** Expression level of each *PYL* gene from the eFP Browser. **(C)** Genomic PCR of *PpPYLs* in the WT and the *PYL* quadruple knockout mutant

pyl1234 qKO. Primer sequences are listed in table S1 and were used for amplification of each *PpPYL* gene using genomic DNA from the WT or *pyl1234 qKO*; the amplified fragments were analyzed by gel electrophoresis. **(D)** RT-PCR analysis of WT and *pyl1234 QKO*. Total RNA was extracted from protonemata without treatment (-) and those treated with 10 µM ABA for 5 h. The reverse transcribed products of each were used for PCR with primer pairs listed in table S1. **(E)** Mutations in *pyl1345 qKO #1* and *#2*. Nucleotide sequences around the positions of genome editing are show for WT and *pyl1345 qKO*. The target sequences are indicated by underlines in the nucleotides of WT. Deleted nucleotides in *pyl1345 qKO* are shown by hyphens, and the altered amino acids are shown in red.

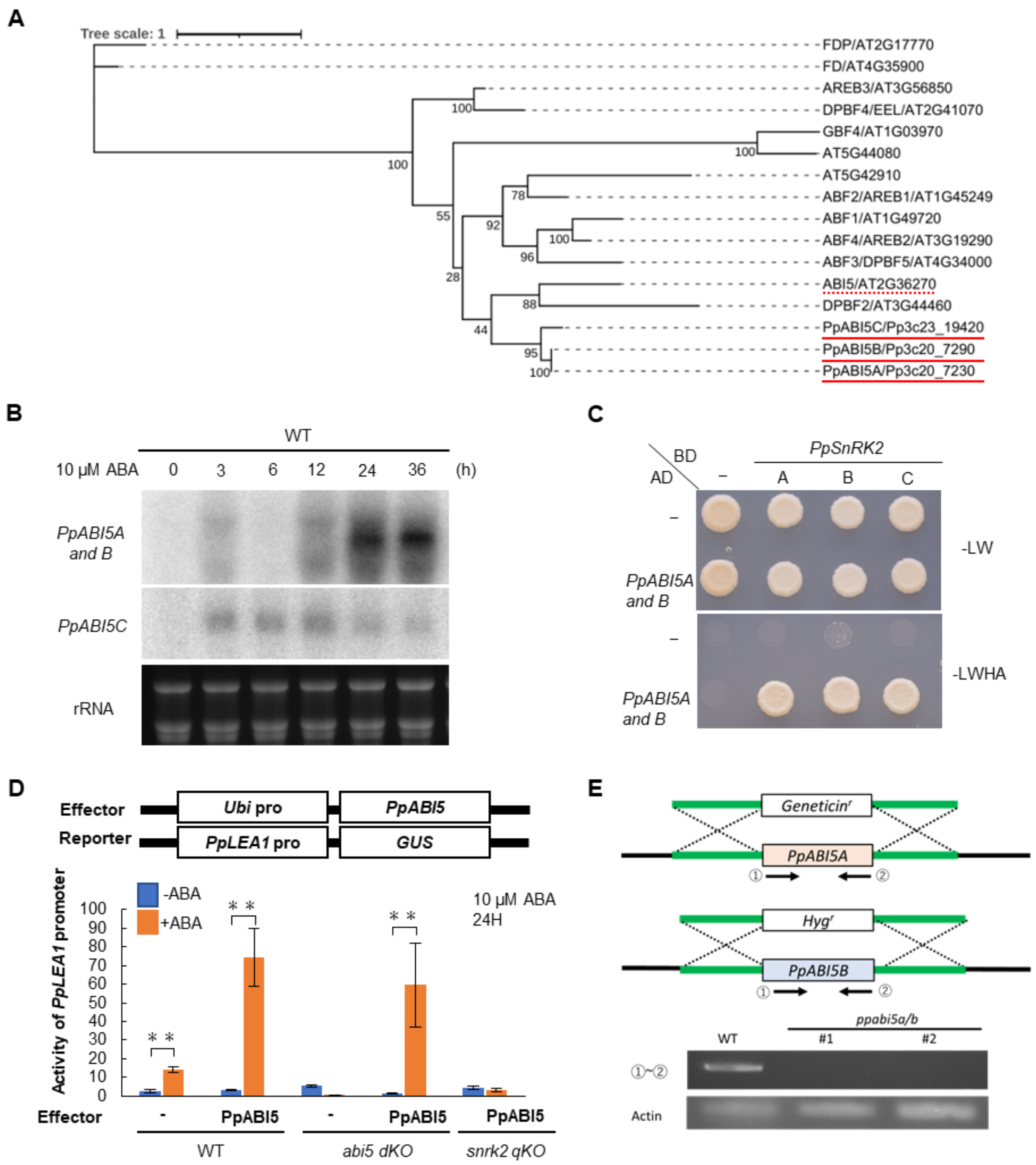

Figure S4.

PpABI5s are ABA-responsive transcription factors acting downstream of subclass III SnRK2. (A) Phylogenetic tree of Group A bZIP-type transcription factors. Numbers on branches denote bootstrap support based on 100 repetitions. Bar, number of amino acid changes per branch length. **(B)** Northern blot of *PpABI5*. Total RNA of protonemal tissues at different time points after ABA treatment was extracted and probed with DNA corresponding to the first exon of *PpABI5A* and *PpABI5B* or the second exon of *PpABI5C*. **(C)** Physical interaction of PpABI5A and PpABI5B, and PpSnRK2s. Physical interaction between PpABI5A (and PpABI5B) and

PpSnRK2s (PpSnRK2A, 2B, and 2C) was investigated using the yeast two-hybrid system. Interaction of the open reading frame of PpABI5A and PpABI5B fused with the GAL4-activation domain (AD) and the open reading frame of PpSnRK2A/B/C fused with the GAL4-binding domain (BD) was assayed. Colonies were grown for 1 week on SD medium lacking leucine and tryptophan (-LW) or lacking leucine, tryptophan, histidine, and adenine (-LWHA); "-" indicates the vector controls. **(D)** Transactivation assay of the *PpLEA1* gene promoter (reporter) by ABI5 in the WT, the double knockout mutant *abi5 dKO*, or the quadruple knockout mutant *snrk2 qKO*. Top: Models of the effector and reporter gene cassettes. Bottom: The effector construct containing the *PpABI5A* gene was introduced into protonemata of the WT (left), *abi5 dKO* (middle), or *snrk2 qKO* (right) with the reporter constructs $PpLEA1_{pro}$-*GUS* and Ubi_{pro}-*LUC*. Protonemal cells were incubated with or without 10 µM ABA for 1 day and then subjected to GUS and LUC assays. As a control, activity of the *PpLEA1* promoter was assessed in the WT, *abi5 dKO* in the absence of the effector construct. Gene expression levels are normalized using the GUS per LUC ratio. Values are mean ± standard error (SE) (n = 3). **$p < 0.01$ compared with −ABA control (Student's t-test). **(E)** Generation of *abi5 dKO lines*. Top: Models of homologous recombination to produce double knockout mutants for *PpABI5A* and *PpABI5B* plants. Bottom: Genomic PCR of *PpABI5A* and *PpABI5B* in the WT and *abi5 dKO*. Since the DNA sequence similarity between *PpABI5A* and *PpABI5B* is high (99.9%), disruption of both *PpABI5A* and *PpABI5B* was confirmed in a single genomic PCR using the same primer set (table S1).

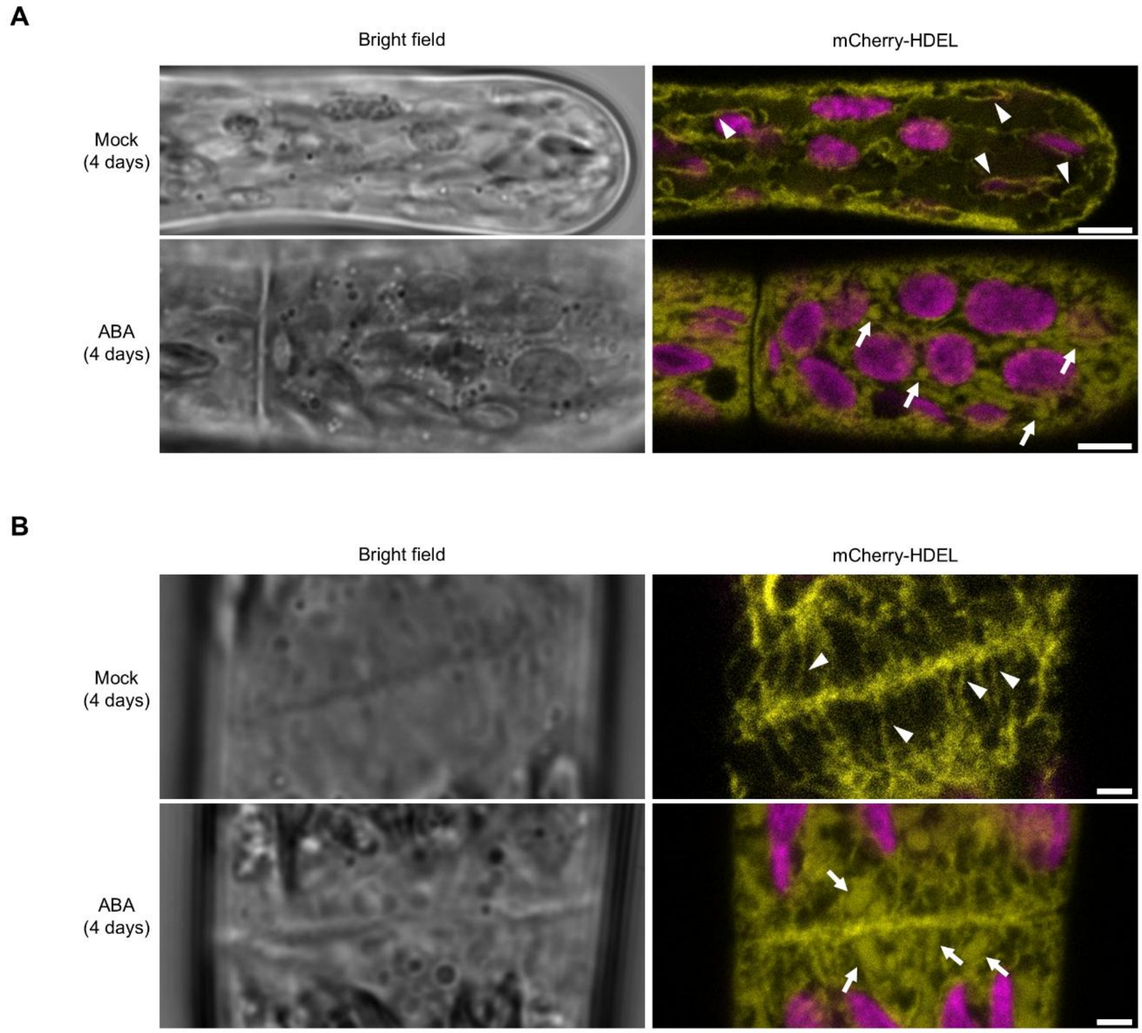

Figure S5.

ER distribution under ABA treatment. (A) Bright field (left) and mCherry-HDEL fluorescence (right) of single plane image in mCherry-HDEL line protonemal cells under 4 days of mock or ABA treatment. Bars, 5 µm. **(B)** Bright field (left) and mCherry-HDEL (right) of cell division plane in apical cells of HDEL-mCherry line. Bars, 2 µm. Magenta colors chloroplast. White arrowheads indicate tubular ER, and white arrows indicate less tubulated ER.

Table S1. Primers used in this study.

Sequence (5' – 3')	Description
CATGTGGGCATAACGATGTACGT	5' primers for PpPYL1 gRNA
AAACACGTACATCGTTATGCCCA	3' primers for PpPYL1 gRNA
CATGCGTGGTGCCGTTGCCTCGG	5' primers for PpPYL2 gRNA
AAACCCGAGGCAACGGCACCACG	3' primers for PpPYL2 gRNA
CATGGATTTCCAGACCCTGCTAG	5' primers for PpPYL3 gRNA
AAACCTAGCAGGGTCTGGAAATC	3' primers for PpPYL3 gRNA
CATGCAGGAGAAACAGGGGCGGC	5' primers for PpPYL4 gRNA
AAACGCCGCCCCTGTTTCTCCTG	3' primers for PpPYL4 gRNA
CATGCGCCCAAGCTCACCCGAGT	5' primers for PpPYL5 gRNA
AAACACTCGGGTGAGCTTGGGCG	3' primers for PpPYL5 gRNA
AGCGTGGATTGACTGCTCTCGC	5' primer of genotype knockout for PpPYL1
TGGCTGGAACACCCGACACCAG	3' primer of genotype knockout for PpPYL1
GAGTGCCAGCAAGTTAGATGCCG	5' primer of genotype knockout for PpPYL2
TCGGATGCGTCGTGGTCGTAGT	3' primer of genotype knockout for PpPYL2
CCAAGAGGCTGCACCGCTTT	5' primer of genotype knockout for PpPYL3
TTGCAACCTGTGGCCTCCTC	3' primer of genotype knockout for PpPYL3
TGCGGAAGATTCGATCGCTATCCG	5' primer of genotype knockout for PpPYL4
TCCAGCCTCTCGATGCTAGATGT	3' primer of genotype knockout for PpPYL4
GAGTGCATTCTTCCTATCCGACGA	5' primer of genotype knockout for PpPYL5
GATTGCATCTCACAACAGTGTCCAC	3' primer of genotype knockout for PpPYL5
ATGAGGTTTGATATTGGGCATA	5' primer of RT-PCR analysis for PpPYL1
CAGGGAGGAGGTTGACAATT	3' primer of RT-PCR analysis for PpPYL1
ATGCAGCAAGTAAAGGGGCGGC	5' primer of RT-PCR analysis for PpPYL2
CCTTTCCATATTTTTATCGG	3' primer of RT-PCR analysis for PpPYL2
ATGCAGACGAAAGGACGTCAAG	5' primer of RT-PCR analysis for PpPYL3
CACTTGCACAGCCTCCTTCT	3' primer of RT-PCR analysis for PpPYL3
TGCGGAAGATTCGATCGCTATCCG	5' primer of RT-PCR analysis for PpPYL4
TCCAGCCTCTCGATGCTAGATGT	3' primer of RT-PCR analysis for PpPYL4
TCTATCTGTCGACTATGGAA	5' primer of RT-PCR analysis for PpTubulin
ATGACATGGATACGCGGGTA	3' primer of RT-PCR analysis for PpTubulin
ATGGCGTCGCGGGCGCTGA	5' primer amplifying DNA probe of PpABI5A/B for northern blot analysis
AACAACAGCAGCAGCAG-3	3' primer amplifying DNA probe of PpABI5A/B for northern blot analysis
TATCGCACAGCAGCAGGCAG	5' primer amplifying DNA probe of PpABI5C for northern blot analysis
GGTCTCGGGCCCGGAAGCAG	3' primer amplifying DNA probe of PpABI5C for northern blot analysis
ggcgcgcccAACATTGCACCTAAAAGAGA	5' primers for PpABI5A 5' homologous recombination
ggcgcgcccGTCCTCCGCCCAAACA	3' primers for PpABI5A 5' homologous recombination
ggcccttatggccAGCGGTGTGGAGGGTGGT	5' primers for PpABI5A 3' homologous recombination
ggccaaatcggccCCTAAACATTTTGGGCTGAAGA	3' primers for PpABI5A 3' homologous recombination
gagcggccgcggccaGGGAATTGCAATGGCCATTGA	5' primers for PpABI5B 5' homologous recombination
taccgtcgacggcccCTCCGTCCTCCGCCCAAACA	3' primers for PpABI5B 5' homologous recombination
agagcggccggcgcgGGCGGTGGGAAATGTGAGA	5' primers for PpABI5B 3' homologous recombination
atgatgcatggcgcgcCTGCCATCCATGAAGAGGTTCA	3' primers for PpABI5B 3' homologous recombination
ATGGCGTCGCGGGCGCTGA	5' primer of genotype knockout for PpABI5A/B
CGAAGACGGCAACGTGGTAG	3' primer of genotype knockout for PpABI5A/B
AACCAATTCAGTCGACATGAAGGTACAGGAGGGT	5' primer for HDEL marker line cloning
AAGCTGGGTCTAGATATCCTTACAGCTCGTCATGAGATCTCTT	3' primer for HDEL marker line cloning
